# Supplementary material for: Genetic, Antigenic, and Pathobiological Characterization of H9 and H6 Low Pathogenicity Avian Influenza Viruses Isolated in Vietnam from 2014 to 2018
Source: Microorganisms. 2023 Jan 18;11(2):244. doi: 10.3390/microorganisms11020244 (PMC9962344; doi:10.3390/microorganisms11020244)
Supplement: Supplementary file 1 [file microorganisms-11-00244-s001.zip › Supplementary Figure S1.pdf]

Supplementary Figure S1A.

N2

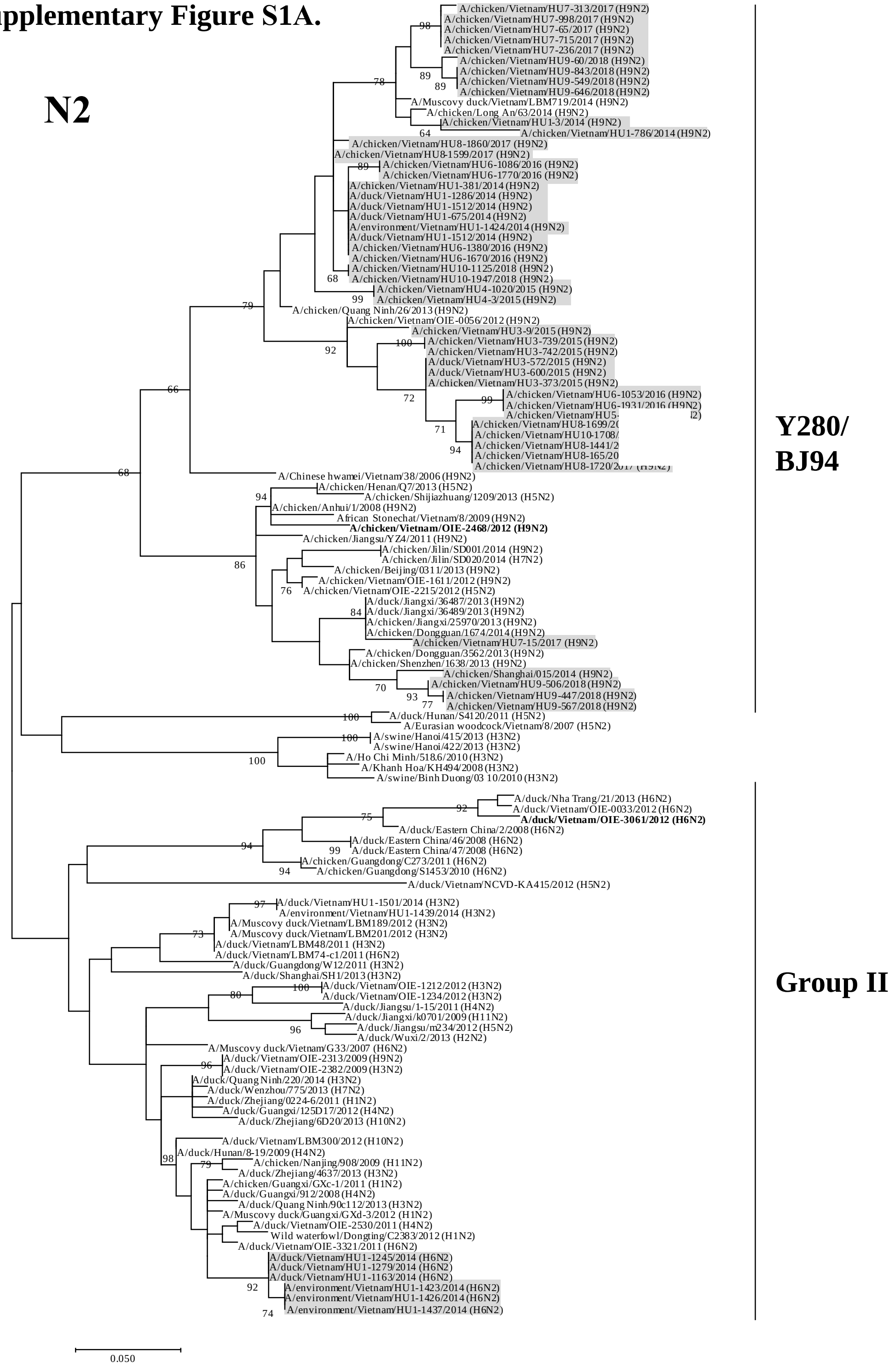

Supplementary Figure S1. Phylogenetic tree of the NA and internal gene segments of H9 and H6 avian influenza viruses. The N2, N6, PB2, PB1, PA, NP, M, and NS genes were used for ML phylogenetic analysis using MEGA 7.0 software. The digits at the nodes indicate the probability of the confidence levels from 1,000 bootstrap replicates. The studied viruses are highlighted in gray and the representative of each sublineage is indicated in bold.

Supplementary Figure S1B.

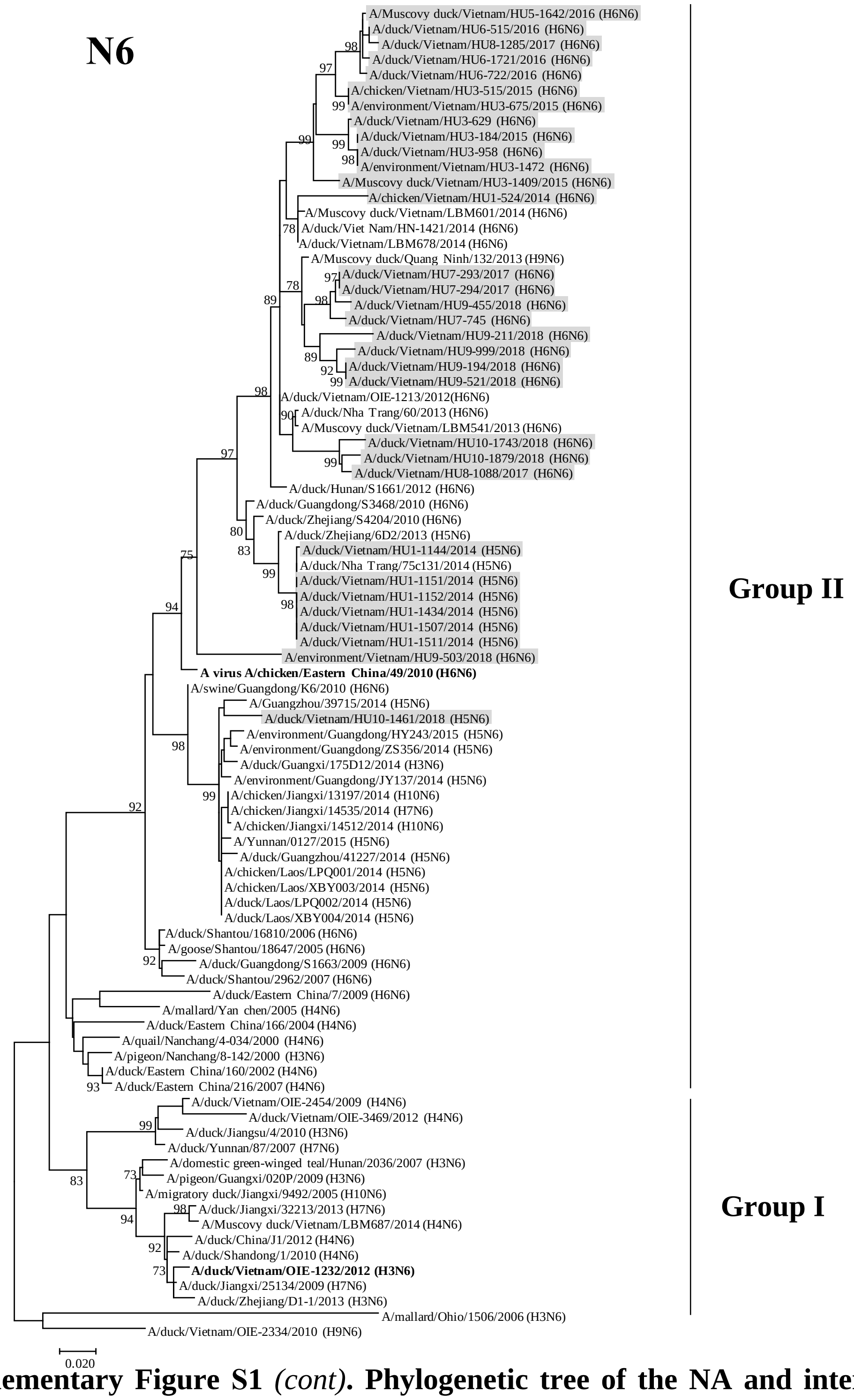

Supplementary Figure S1 ***(cont)*****. Phylogenetic tree of the NA and internal gene segments of H9 and H6 avian influenza viruses.** The N2, N6, PB2, PB1, PA, NP, M, and NS genes were used for ML phylogenetic analysis using MEGA 7.0 software. The digits at the nodes indicate the probability of the confidence levels from 1,000 bootstrap replicates. The studied viruses are highlighted in gray and the representative of each sublineage is indicated in bold.

Supplementary Figure S1C.

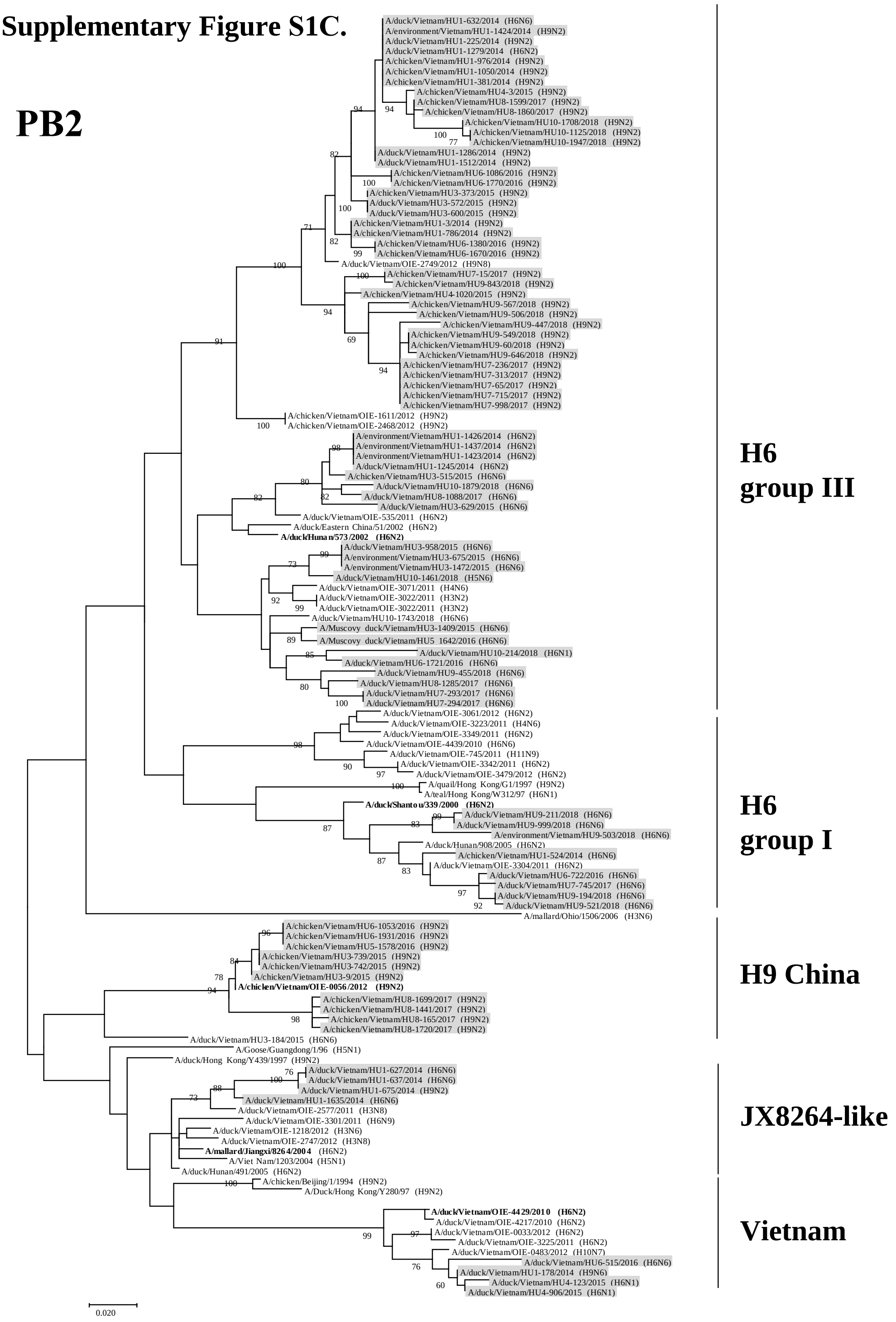

Supplementary Figure S1 *(cont)*. **Phylogenetic tree of the NA and internal gene segments of H9 and H6 avian influenza viruses.** The N2, N6, PB2, PB1, PA, NP, M, and NS genes were used for ML phylogenetic analysis using MEGA 7.0 software. The digits at the nodes indicate the probability of the confidence levels from 1,000 bootstrap replicates. The studied viruses are highlighted in gray and the representative of each sublineage is indicated in bold.

Supplementary Figure S1D.

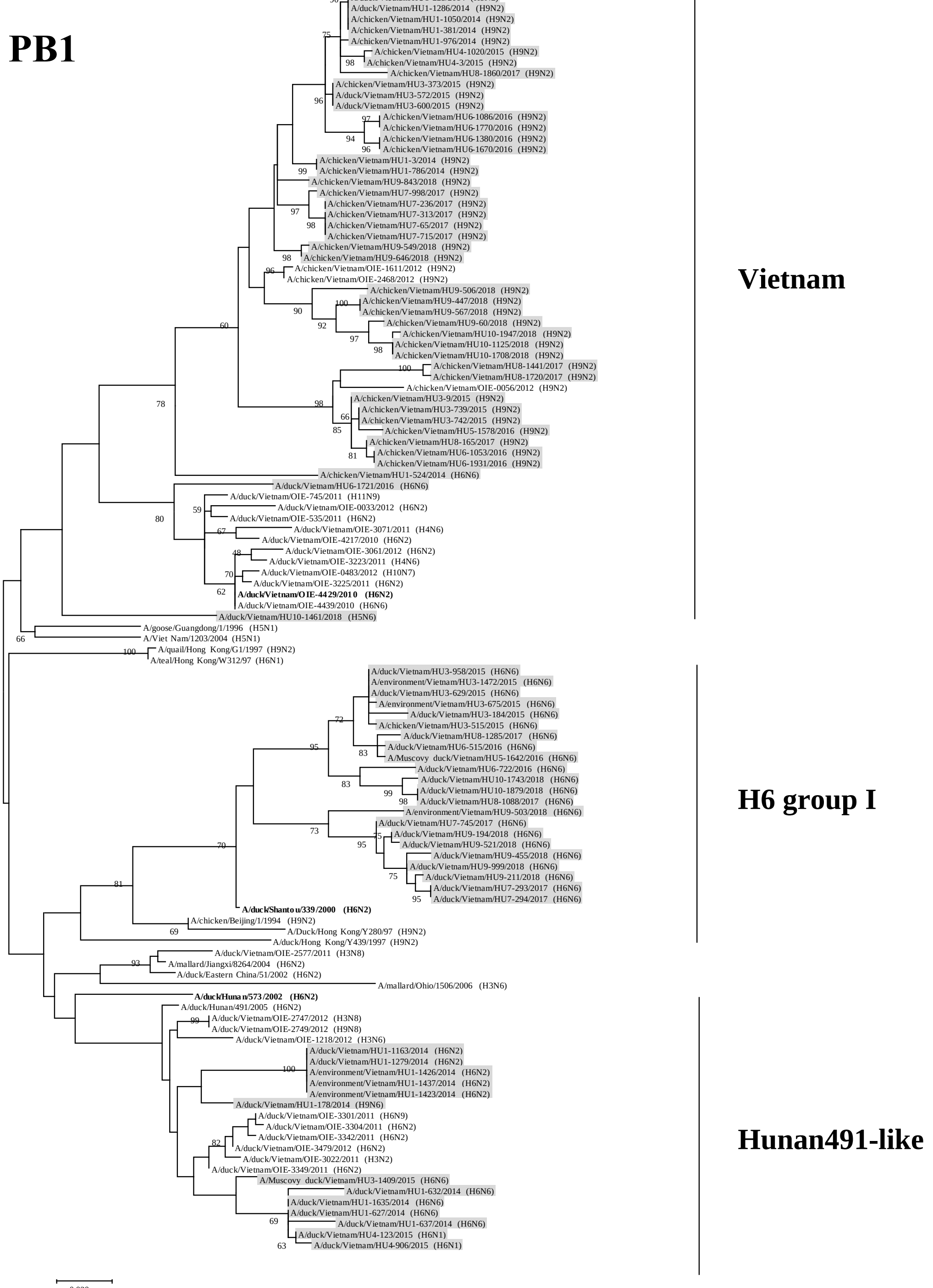

Supplementary Figure S1 *(cont)*. **Phylogenetic tree of the NA and internal gene segments of H9 and H6 avian influenza viruses.** The N2, N6, PB2, PB1, PA, NP, M, and NSgenes were used for ML phylogenetic analysis using MEGA 7.0 software. The digits at the nodes indicate the probability of the confidence levels from 1,000 bootstrap replicates. The studied viruses are highlighted in gray and the representative of each sublineage is indicated in bold.

Supplementary Figure S1E.

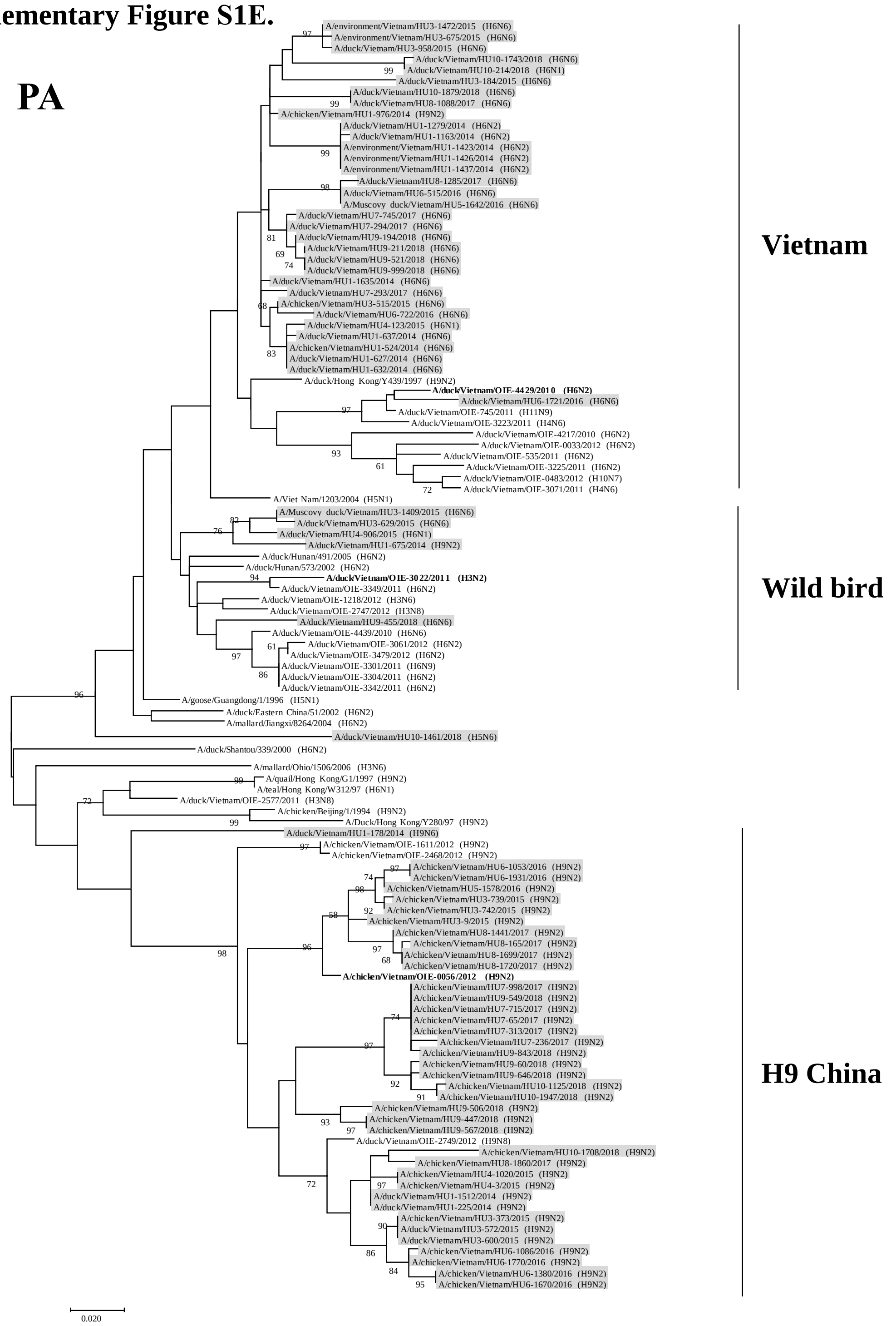

Supplementary Figure S1 *(cont)*. **Phylogenetic tree of the NA and internal gene segments of H9 and H6 avian influenza viruses.** The N2, N6, PB2, PB1, PA, NP, M, and NS genes were used for ML phylogenetic analysis using MEGA 7.0 software. The digits at the nodes indicate the probability of the confidence levels from 1,000 bootstrap replicates. The studied viruses are highlighted in gray and the representative of each sublineage is indicated in bold.

Supplementary Figure S1F.

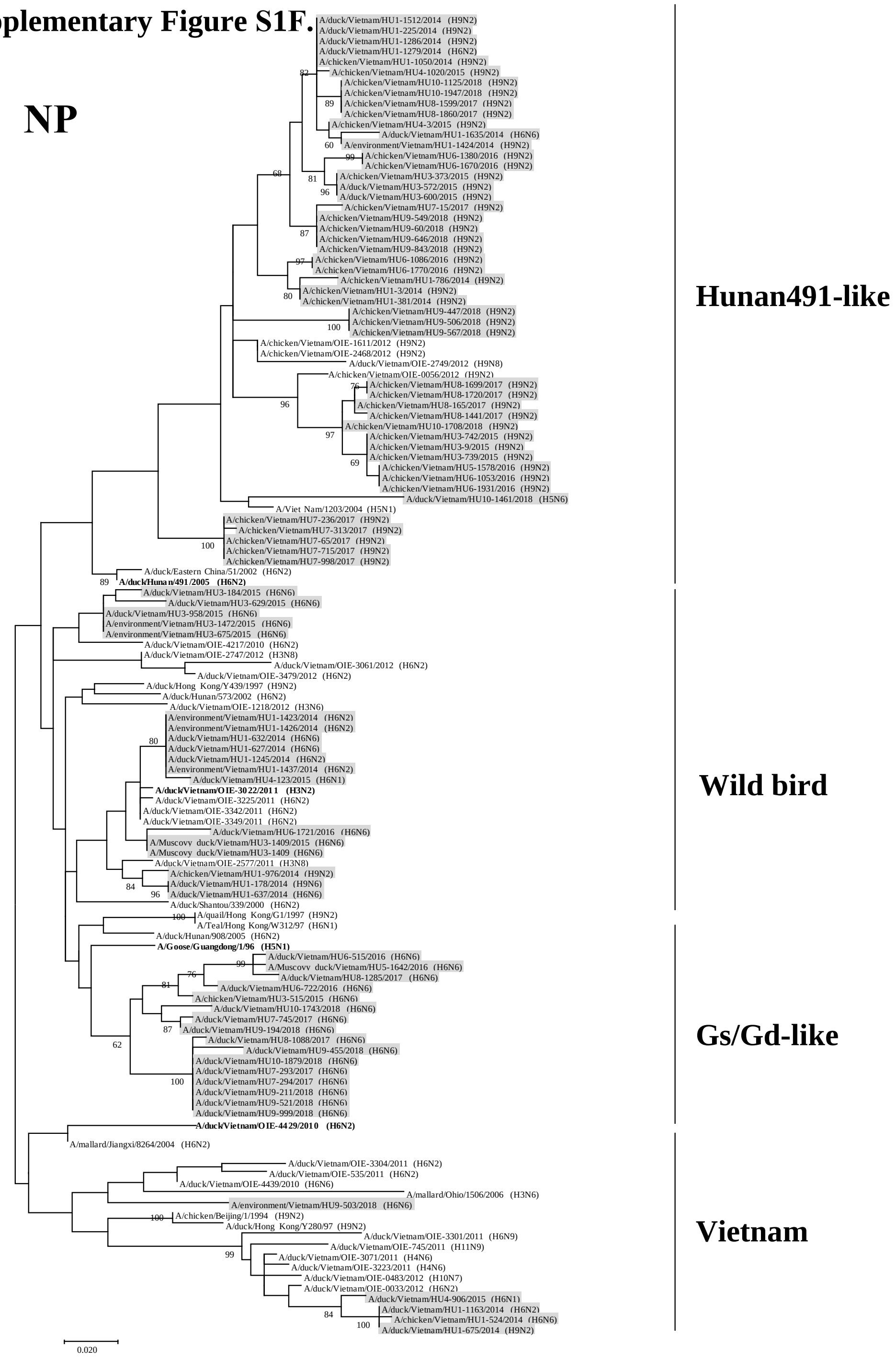

Supplementary Figure S1 *(cont)*. **Phylogenetic tree of the NA and internal gene segments of H9 and H6 avian influenza viruses.** The N2, N6, PB2, PB1, PA, NP, M, and NSgenes were used for ML phylogenetic analysis using MEGA 7.0 software. The digits at the nodes indicate the probability of the confidence levels from 1,000 bootstrap replicates. The studied viruses are highlighted in gray and the representative of each sublinge is indicated in bold.

Supplementary Figure S1G.

M

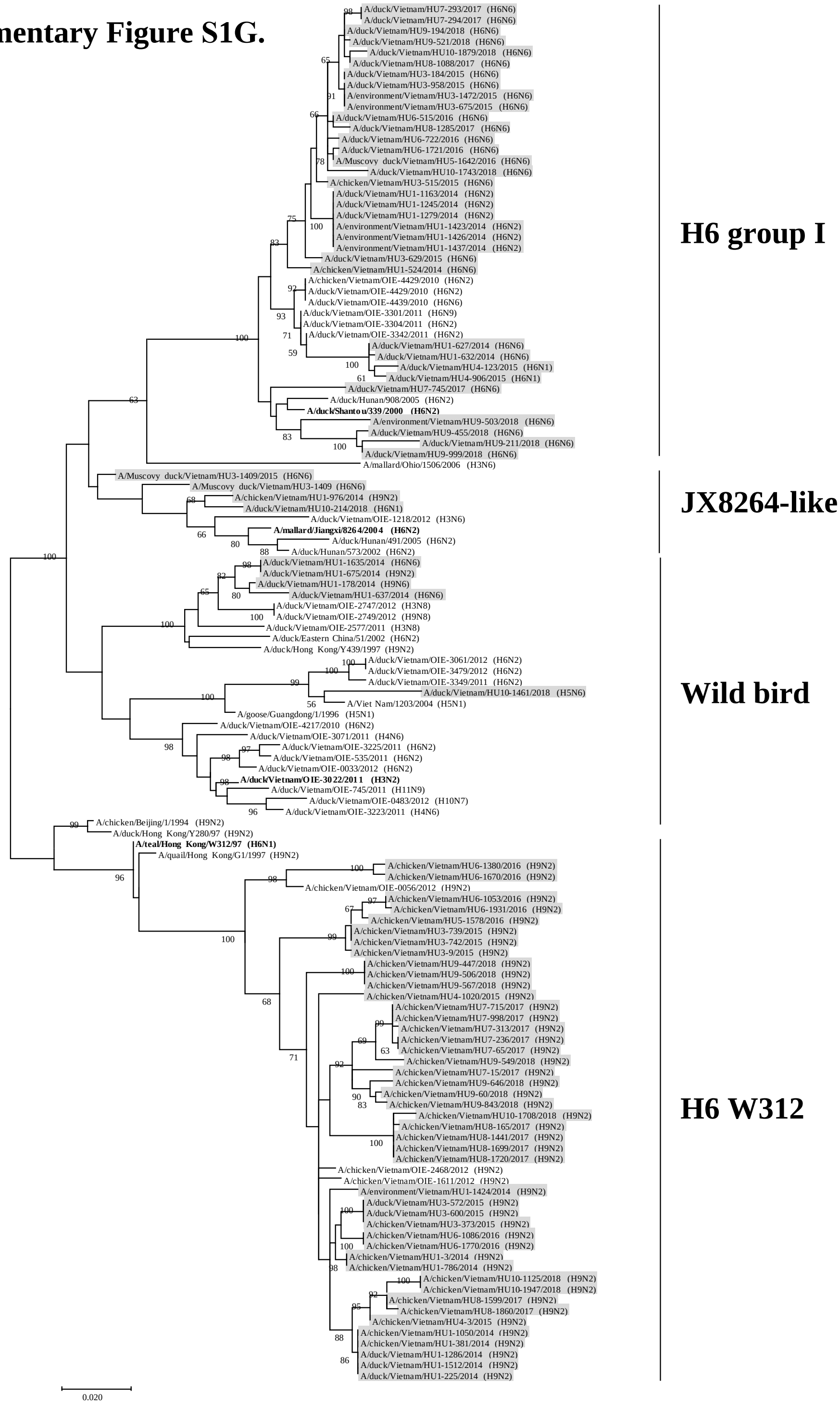

Supplementary Figure S1 ***(cont)*****. Phylogenetic tree of the NA and internal gene segments of H9 and H6 avian influenza viruses.** The N2, N6, PB2, PB1, PA, NP, M, and NSgenes were used for ML phylogenetic analysis using MEGA 7.0 software. The digits at the nodes indicate the probability of the confidence levels from 1,000 bootstrap replicates. The studied viruses are highlighted in gray and the representative of each sublineage is indicated in bold.

Supplementary Figure S1H.

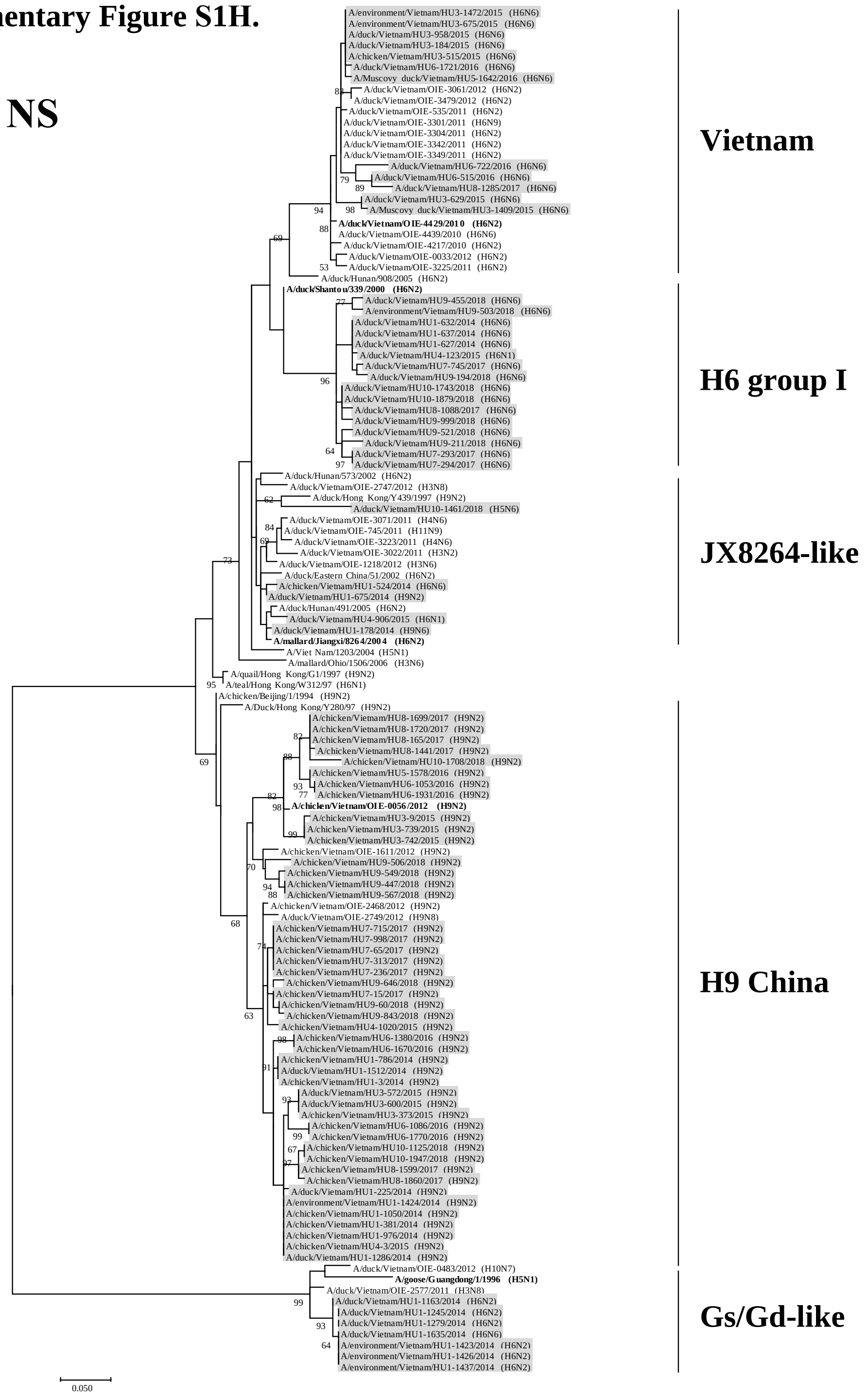

Supplementary Figure S1 ***(cont)*****. Phylogenetic tree of the NA and internal gene segments of H9 and H6 avian influenza viruses.** The N2, N6, PB2, PB1, PA, NP, M, and NS genes were used for ML phylogenetic analysis using MEGA 7.0 software. The digits at the nodes indicate the probability of the confidence levels from 1,000 bootstrap replicates. The studied viruses are highlighted in gray and the representative of each sublineage is indicated in bold.
