# Supplementary material for: Genetic, Antigenic, and Pathobiological Characterization of H9 and H6 Low Pathogenicity Avian Influenza Viruses Isolated in Vietnam from 2014 to 2018
Source: Microorganisms. 2023 Jan 18;11(2):244. doi: 10.3390/microorganisms11020244 (PMC9962344; doi:10.3390/microorganisms11020244)
Supplement: Supplementary file 1 [file microorganisms-11-00244-s001.zip › Supplementary Figure S2.pdf]

Supplementary Figure S2.

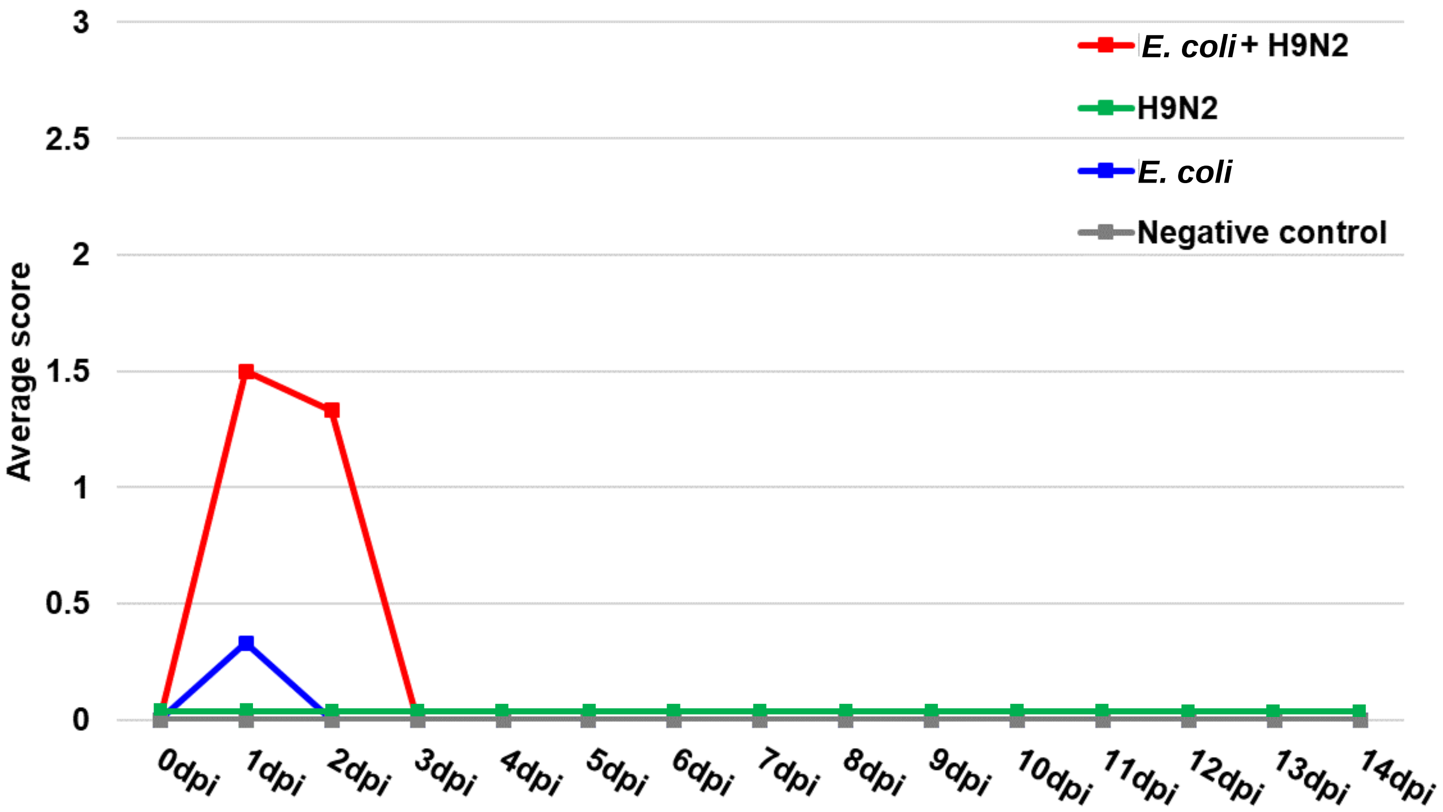

Supplementary Figure S2. Clinical scores in different groups of chickens (n = 6) after inoculation with H9 LPAIV (A/chicken/Vietnam/HU8-1860/2017) or *E. coli* O2 or a combination of both. The score was recorded every day from 1 dpi to 14 dpi. The clinical signs were scored on the following scale: 0 - no sign, 1 - sick, 2 - severely sick, 3 - dead.
