# Supplementary material for: Genetic, Antigenic, and Pathobiological Characterization of H9 and H6 Low Pathogenicity Avian Influenza Viruses Isolated in Vietnam from 2014 to 2018
Source: Microorganisms. 2023 Jan 18;11(2):244. doi: 10.3390/microorganisms11020244 (PMC9962344; doi:10.3390/microorganisms11020244)
Supplement: Supplementary file 1 [file microorganisms-11-00244-s001.zip › Supplementary Table S1.pdf]

Supplementary Table S1. Summary of AIV surveillance in Vietnam from 2014 to 2018

Year	Region	Province	No. of samples	AIV positive	Prevalence (%) (95% CI)	Subtype (no. of isolates)	Reference
2014	Central	Hue	3,045	178	5.8 (5.0–6.7)	H3N2 (18), H3N6 (1), H4N6 (2), **H5N6 (8)**, H6N2 (14), H6N6 (16), H9N2 (109), H9N6 (5), H11N6 (1), H11N7 (4)	Chu *et al.* 2016 Chu *et al.* 2017
2015	Central	Hue	2,040	49	2.4 (1.8–3.2)	H3N1 (1), H3N8 (3), H4N2 (3), **H5N1 (4)**, **H5N6 (9)**, H6N1 (14), H9N2 (15)	Nguyen *et al.* 2019
	South	Vinh Long	1,400	243	17.4 (15.4–19.4)	H3N2 (1), H4N6 (1), **H5N1 (130)**, H6N6 (24), H9N2 (86), H11N9 (1)	Nguyen *et al.* 2019
2016	South	Vinh Long	3,300	131	4.0 (3.3–4.7)	H3N2 (11), H3N8 (2), **H5N1 (5)**, H6N6 (69), H9N2 (31), H10N6 (7), H11N9 (5), H12N5 (1)	Nguyen *et al.* 2019; Le *et al.* 2021
2017	North	Lang Son	1,000	148	14.8 (12.7–17.2)	**H5N6 (6)**, H6N6 (3), H9N2 (139)	Nguyen *et al.* 2019
	South	Vinh Long	1,800	167	9.3 (8.0–10.7)	H3N2 (2), **H5N1 (21)**, H6N6 (63), H9N2 (79), H10N3 (2)	Nguyen *et al.* 2019; Le *et al.* 2021
2018	North	Lang Son	1,000	306	30.6 (27.8–33.6)	H3N2 (29), **H5N6 (2)**, H6N6 (89), H9N2 (186)	This study
	South	Vinh Long	1,846	139	7.5 (6.4–8.8)	H3N2 (1), H4N6 (2), **H5N1 (17)**, **H5N6 (11)**, H6N6 (52), H7N7 (3), H9N2 (47), H9N6 (1), H11N1 (1), H11N9 (3), H13N9 (1)	Le *et al.* 2020
Total			15,431	1,361	8.8 (8.4–9.3)		

CI: confidence interval

High pathogenicity avian influenza viruses are highlighted in bold.
