# Supplementary material for: Genetic, Antigenic, and Pathobiological Characterization of H9 and H6 Low Pathogenicity Avian Influenza Viruses Isolated in Vietnam from 2014 to 2018
Source: Microorganisms. 2023 Jan 18;11(2):244. doi: 10.3390/microorganisms11020244 (PMC9962344; doi:10.3390/microorganisms11020244)
Supplement: Supplementary file 1 [file microorganisms-11-00244-s001.zip › Supplementary Table S2.pdf]

Supplementary Table S2. Primers used to detect virulence-associated genes and the results of virulence-associated gene detection in avian pathogenic *E. coli* (APEC) strains isolated in this study

Target gene	Primer	Primer sequence	Amplicon size (bp)	*E. coli* strain				Reference
				O1	O2	O18	O78	
papC	papC-F<br>papC-R	TGATATCACGCAGTCAGTAGC<br>CCGGCCATATTCACATAA	500	-	-	-	-	Janßen *et al.* 2001
fimC	fimC-F<br>fimC-R	GGGTAGAAAATGCCGATGGTG<br>CGTCATTTTGGGGGTAAGTGC	496	+	+	+	+	
tsh	tsh-F<br>tsh-R	GTGATAAACAAGTCGGCAACA<br>GCATTGAGACATCCATTCC	804	-	+	-	-	
iucD	iucD-F<br>iucD-R	ACAAAAAGTTCTATCGCTTCC<br>CCTGATCCAGATGATGCTC	692	+	+	+	-	
astA	astA-F<br>astA-R	TGCCATCAACACAGTATATCC<br>TCAGGTCGCGAGTGACGGC	116	+	+	+	+	Subedi *et al.* 2018
iutA	iutA-F<br>iutA-R	GGCTGGACATCATGGGAACTGG<br>CGTCGGGAACGGGTAGAATCG	302	+	+	+	+	
irp2	irp2-F<br>irp2-R	AAGGATTCGCTGTTACCGGAC<br>AACTCCTGATACAGGTGGC	413	+	+	+	+	
cva/cviC	cva/cvi-F<br>cva/cvi-R	TCCAAGCGGACCCCTTATAG<br>CGCAGCATAGTTCCATGCT	598	-	-	-	-	Prioste *et al.* 2013
iss	iss-F<br>iss-R	GTGGCGAAAACTAGTAAAACAGC<br>CGCCTCGGGGTGGATAA	760	+	+	+	+	Horne *et al.* 2000
