# Supplementary material for: Genetic, Antigenic, and Pathobiological Characterization of H9 and H6 Low Pathogenicity Avian Influenza Viruses Isolated in Vietnam from 2014 to 2018
Source: Microorganisms. 2023 Jan 18;11(2):244. doi: 10.3390/microorganisms11020244 (PMC9962344; doi:10.3390/microorganisms11020244)
Supplement: Supplementary file 1 [file microorganisms-11-00244-s001.zip › Supplementary Table S3.pdf]

Supplementary Table S3. Antigenic analyses of H9 influenza viruses using cross-HI test

Lineage	Sub lineage	Viruses	HI titers of the antisera				
			Eurasian				North American
			Y280/BJ94		G1	Y439	
			HK/Y280/97	VN/1860/17	HK/G1/97	Hok/49/98	Wis/1/66
Eurasian	Y280/BJ94	A/Duck/Hong Kong/Y280/1997 (H9N2)	20,480	10,240	1,280	5,120	640
		A/chicken/Vietnam/OIE-1611/2012 (H9N2)	10,240	10,240	1,280	2,560	320
		**A/chicken/Vietnam/HU1-786/2014 (H9N2)**	10,240	10,240	1,280	5,120	320
		**A/chicken/Vietnam/HU3-742/2015 (H9N2)**	10,240	10,240	1,280	5,120	640
		**A/chicken/Vietnam/HU7-236/2017 (H9N2)**	20,480	10,240	1,280	5,120	320
		**A/chicken/Vietnam/HU8-1860/2017 (H9N2)**	20,480	10,240	2,560	10,240	1,280
	G1	A/quail/Hong Kong/G1/1997 (H9N2)	2,560	640	10,240	1,280	640
		A/duck/Vietnam/OIE-2592/2009 (H9N2)	10,240	1,280	5,120	5,120	640
	Y439	A/duck/Hokkaido/49/1998 (H9N2)	320	80	80	2,560	320
		A/duck/Vietnam/OIE-2334/2010 (H9N6)	160	40	160	1,280	320
North American	-	A/turkey/Wisconsin/1/1966 (H9N2)	80	40	40	1,280	2,560

Viruses isolated in this study are highlighted in bold.

Homologous titers are underlined.

Dk duck, Ck chicken, Ty Turkey, Hok Hokkaido, HK Hong Kong, Wis Wisconsin.
