# Supplementary material for: Genetic, Antigenic, and Pathobiological Characterization of H9 and H6 Low Pathogenicity Avian Influenza Viruses Isolated in Vietnam from 2014 to 2018
Source: Microorganisms. 2023 Jan 18;11(2):244. doi: 10.3390/microorganisms11020244 (PMC9962344; doi:10.3390/microorganisms11020244)
Supplement: Supplementary file 1 [file microorganisms-11-00244-s001.zip › Supplementary Table S4.pdf]

**Supplementary Table S4.** Antigenic analyses of H6 influenza viruses using cross-HI test

| Lineage | Sub lineage | Virus | HI titers of the antiserum | | | | | | | |
|---|---|---|---|---|---|---|---|---|---|---|
| | | | Eurasian | | | | | | | North American |
| | | | Early | W312 | Group II | | | Group III | | |
| | | | HK/960 /1980 | HK/W3 12/1997 | VN/OIE -4429 /2010 | VN/HU1 -637 /2014 | VN/HU8 -1088 /2017 | Hok/262/ 2004 | Aus/1 /1972 | Mass/3740 /1965 |
| Eurasia | Early | A/duck/Hong Kong/960/1980 (H6N2) | 5,120 | 160 | 160 | 320 | 1,280 | 2,560 | 640 | 160 |
| | W312 | A/teal/Hong Kong/W312/1997 (H6N1) | 2,560 | 1,280 | 40 | 320 | 640 | 320 | 160 | 160 |
| | Group II | A/duck/Vietnam/OIE-4429/2010 (H6N2) | 2,560 | 80 | 5,120 | 1,280 | 2,560 | 160 | 80 | 160 |
| | | **A/duck/Vietnam/HU1-1245/2014 (H6N2)** | 640 | 80 | 640 | 20,480 | 20,480 | 320 | 80 | 80 |
| | | **A/duck/Vietnam/HU1-637/2014 (H6N6)** | 640 | 80 | 320 | 20,480 | 10,240 | 160 | 40 | 20 |
| | | **A/duck/Vietnam/HU3-629/2015 (H6N6)** | 160 | 20 | 160 | 2,560 | 5,120 | 160 | 20 | 20 |
| | | **A/duck/Vietnam/HU6-1721/2016 (H6N6)** | 320 | 40 | 160 | 10,240 | 10,240 | 80 | 40 | <20 |
| | | **A/duck/Vietnam/HU7-745/2017 (H6N6)** | 80 | 20 | 160 | 1,280 | 1,280 | 40 | <20 | <20 |
| | | **A/duck/Vietnam/HU8-1088/2017 (H6N6)** | 640 | 20 | 80 | 5,120 | 10,240 | 80 | 20 | <20 |
| | | **A/duck/Vietnam/HU9-455/2018 (H6N6)** | 640 | 20 | 80 | 5,120 | 10,240 | 80 | 20 | <20 |
| | | **A/duck/Vietnam/HU10-1879/2018 (H6N6)** | 160 | 20 | 160 | 5,120 | 10,240 | 80 | <20 | <20 |
| | Group III | A/duck/Hokkaido/262/2004 (H6N1) | 5,120 | 160 | 160 | 160 | 2,560 | 2,560 | 1,280 | 160 |
| | | **A/duck/Vietnam/HU4-906/2015 (H6N6)** | 2,560 | 160 | 5,120 | 640 | 2,560 | 320 | 80 | 80 |
| | - | A/shearwater/Australia/1/1972 (H6N5) | 2,560 | 320 | 160 | 160 | 640 | 1,280 | 1,280 | 320 |
| North American | | A/turkey/Massachusetts/3740/1965 (H6N2) | 2,560 | 320 | 160 | 160 | 2,560 | 640 | 1,280 | 640 |

Viruses isolated in this study are highlighted in bold.
Homologous titers are underlined.
Dk duck, Ck chicken, Ty Turkey, Tl Teal, Sh Shearwater, Hok Hokkaido, HK Hong Kong, Aus Australia, Mass Massachusetts.
